# Improvement of Uveal and Capsular Biocompatibility of Hydrophobic Acrylic Intraocular Lens by Surface Grafting with 2-Methacryloyloxyethyl Phosphorylcholine-Methacrylic Acid Copolymer

**DOI:** 10.1038/srep40462

**Published:** 2017-01-13

**Authors:** Xuhua Tan, Jiezhao Zhan, Yi Zhu, Ji Cao, Lin Wang, Sa Liu, Yingjun Wang, Zhenzhen Liu, Yingyan Qin, Mingxing Wu, Yizhi Liu, Li Ren

**Affiliations:** 1 State Key Laboratory of Ophthalmology, Zhongshan Ophthalmic Center, Sun Yat-sen University, Guangzhou, Guangdong, 510060, China; 2 National Engineering Research Center for Human Tissue Restoration and Reconstruction, South China University of Technology, Guangzhou, Guangdong, 510006, China; 3 School of Materials Science and Engineering, South China University of Technology, Guangzhou, Guangdong, 510641, China; 4 EYEGOOD Medicals Co., Ltd, Zhuhai, Guangdong, 519085, China; 5 Zhongshan Ophthalmic Center, Sun Yat-sen University 54 South Xianlie Rd, Guangzhou, China; 6 Department of Cataract, Zhongshan Ophthalmic Center, Sun Yat-sen University, Guangzhou, Guangdong, 510060, China

## Abstract

Biocompatibility of intraocular lens (IOL) is critical to vision reconstruction after cataract surgery. Foldable hydrophobic acrylic IOL is vulnerable to the adhesion of extracellular matrix proteins and cells, leading to increased incidence of postoperative inflammation and capsule opacification. To increase IOL biocompatibility, we synthesized a hydrophilic copolymer P(MPC-MAA) and grafted the copolymer onto the surface of IOL through air plasma treatment. X-ray photoelectron spectroscopy, atomic force microscopy and static water contact angle were used to characterize chemical changes, topography and hydrophilicity of the IOL surface, respectively. Quartz crystal microbalance with dissipation (QCM-D) showed that P(MPC-MAA) modified IOLs were resistant to protein adsorption. Moreover, P(MPC-MAA) modification inhibited adhesion and proliferation of lens epithelial cells (LECs) *in vitro*. To analyze uveal and capsular biocompatibility *in vivo*, we implanted the P(MPC-MAA) modified IOLs into rabbits after phacoemulsification. P(MPC-MAA) modification significantly reduced postoperative inflammation and anterior capsule opacification (ACO), and did not affect posterior capsule opacification (PCO). Collectively, our study suggests that surface modification by P(MPC-MAA) can significantly improve uveal and capsular biocompatibility of hydrophobic acrylic IOL, which could potentially benefit patients with blood-aqueous barrier damage.

Cataract is the leading cause of vision loss worldwide, accounting for 11 million cases of blindness and 35 million cases of visual impairment per year^1^. So far, phacoemulsification with foldable intraocular lens (IOL) implantation is the main treatment for cataract. Foldable hydrophobic acrylic IOLs are most commonly used due to the high refractive index and relatively low posterior capsule opacification (PCO) rate^2^. However, extracellular matrix proteins, inflammatory cells and lens epithelial cells (LECs) can easily adhere to the hydrophobic surface, leading to a high incidence of iris posterior synechiae (IPS) and anterior capsule opacification (ACO)^3^,^4^,^5^,^6^, especially in patients with blood-aqueous barrier damage. Serious ACO may cause anterior capsule shrinkage, IOL decentration, and may hinder the examination of peripheral fundus^7^. These problems limit the application of hydrophobic acrylic IOL in patients with uveitis, glaucoma or diabetes.

Improvement of IOL biocompatibility, including uveal and capsular biocompatibility, is critical to reduce postoperative complications^8^. Uveal biocompatibility is the foreign-body reaction to the IOL implant. Disruption of the blood-aqueous barrier (BAB) in cataract surgery leads to the release of protein and inflammatory cells into the aqueous humor^9^. Also, the implanted IOL directly interacts with the surrounding tissue, activates the complement system, and triggers inflammation of iris, ciliary body and anterior choroid^10^. Capsular biocompatibility refers to the interaction between the IOL and LECs. Once the lens is removed, remnant LECs in the capsule can attach to the IOL surface, and then proliferate, migrate and differentiate into fibroblast-like cells, causing ACO and PCO^8^,^11^. Moreover, inflammatory cells such as macrophages and monocytes secrete growth factors and cytokines that may aggravate capsule opacification^12^.

One way to improve IOL biocompatibility is to reduce adhesion of inflammatory cells and LECs onto the IOL surface. The initial phase of cell adhesion is the interaction between cell surface adhesion molecules and extracellular matrix (ECM) proteins, such as fibronectin, laminin and collagen^13^. Many studies have demonstrated that hydrophilic surface can reduce protein adsorption and cell adhesion, and surface modifications of hydrophobic acrylic IOL have been attempted to increase biocompatibility without changing the bulk properties^14^,^15^,^16^,^17^. 2-methacryloyloxyethyl phosphorylcholine (MPC, Supplementary Fig. S1a) is a zwitterionic molecule and shows excellent biocompatibility since it can form a membrane-like structure and trap water molecules on the material surface, suppressing protein adsorption and cell adhesion^14^,^18^,^19^,^20^. Surface modification by MPC has been widely applied in bioimplants, tissue engineering and drug delivery systems^21^,^22^,^23^. Surface modification of silicone IOL by MPC can significantly reduce water contact angle, and suppress adhesion of epithelial and fibroblast-like cells^14^,^18^. However, irreversible trap of silicone oil limits the application of silicone IOL in patients with vitreoretinal diseases^24^. Therefore, silicone IOLs are not widely used as hydrophobic acrylic IOLs in cataract surgery. In acrylic IOL, MPC coating suppresses fibroblast and bacterial adhesion *in vitro*^25^. However, physisorbed surface coating is less stable than covalent binding. Also, uveal and capsular biocompatibility of MPC-modified hydrophobic acrylic IOL has not been evaluated *in vivo*.

MPC modification on hydrophobic silicone IOL did not reduce aqueous flare in a rabbit model^14^, suggesting that grafting MPC monomers alone is insufficient to alleviate post-operative anterior chamber inflammation. One possible explanation is that MPC does not carry enough negative charges to sufficiently reduce protein adsorption and cell adhesion as extracellular protein is usually negatively charged under physiological conditions^16^,^17^,^26^. Methyl acrylic acid (MAA, Supplementary Fig. S1b) is a negatively charged hydrophilic monomer due to the carboxylic acid group^27^, and has been applied to resist cell invasion and prevent tissue adhesions after surgery^19^. MAA has been widely used in the fabrication of contact lens hydrogels and showed excellent biocompatibility^28^,^29^. Here, to combine the advantages of MPC and MAA together, we used MPC and MAA to synthesize a hydrophilic copolymer P(MPC-MAA) (Supplementary Fig. S1c), and covalently grafted this copolymer onto the surface of hydrophobic acrylic IOL after ammonia plasma treatment. Then we characterized the IOL surface property and evaluated the uveal and capsular biocompatibility of P(MPC-MAA)-modified hydrophobic acrylic IOL both *in vitro* and *in vivo*.

## Results

### P(MPC-MAA) was synthesized and grafted onto the IOL surface

**Figure 1:**
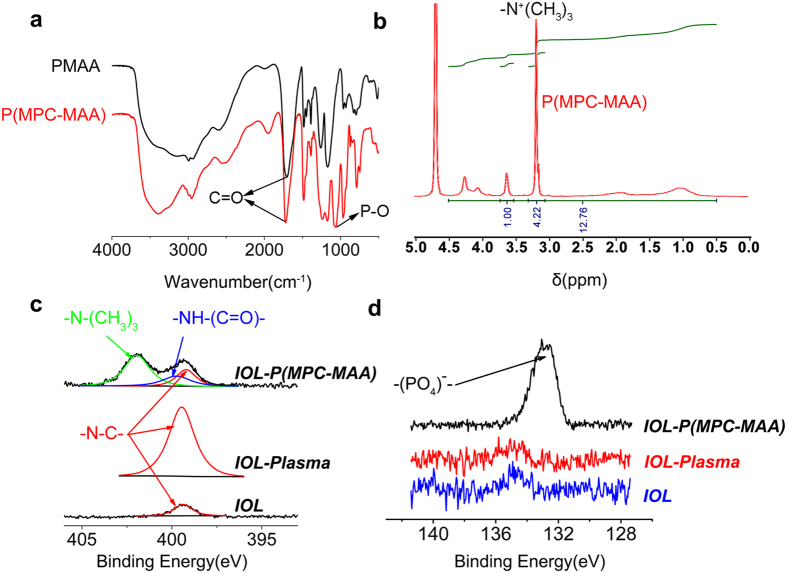
P(MPC-MAA) was successfully synthesized and grafted onto the IOL surface. (**a**) FT-IR spectra of PMAA and P(MPC-MAA) detecting with the potassium bromide pressed-disk technique for 32 scans over the 500–4,000 cm^−1^ range at a resolution of 4.0 cm^−1^. (**b**) H-NMR spectra of P(MPC-MAA) determined at 400 MHz. (**c**,**d**) N1s high-resolution spectra and P1s high-resolution spectra of ***IOL**, **IOL-Plasma***, and ***IOL-P***(***MPC-MAA**)*, respectively.

P(MPC-MAA) copolymer was synthetized via free radical polymerization. Fourier transform infrared (FT-IR) spectroscopy and proton nuclear magnetic resonance (^1^H NMR) spectroscopy of P(MPC-MAA) are shown in Fig. 1. A transmission absorption peak was observed at 1,720 cm^−1^ for all of the samples (Fig. 1a), which corresponded to the carbonyl group (C=O) in the PMAA and P(MPC-MAA). However, an absorption peak at 1,080 cm^−1^ was observed only in the spectra for P(MPC-MAA), which corresponded to the phosphate group (P-O) in the MPC unit^30^,^31^. The proton signals at 3.2 ppm were observed in the ^1^H NMR spectroscopy of P(MPC-MAA) (Fig. 1b), which was attributed to -N^+^(CH_3_)_3_ of the MPC units^30^,^32^,^33^. Collectively, these results demonstrated that the P(MPC-MAA) copolymer was successfully synthesized. The molar fractions of MPC and MAA were 5.7:4.3 calculated from ^1^H NMR spectroscopy. The Molecular weight (Mw) and polydispersity index (PDI, Mw/Mn) of P(MPC-MAA) copolymer were 2.3 × 10^5^ and 3.02, respectively (Supplementary Fig. S2).

To construct a protein-resistant IOL surface, P(MPC-MAA) copolymer was grafted onto the IOL surface via plasma technology. Untreated hydrophobic IOL, IOL treated by plasma alone, and P(MPC-MAA) modified IOL are abbreviated as ***IOL**, **IOL-Plasma*** and ***IOL-P***(***MPC-MAA**)*, respectively. X-ray photoelectron spectroscopy (XPS) spectra of the binding energy regions of the nitrogen (N) and phosphorous (P) electrons of ***IOL**, **IOL-Plasma*** and ***IOL-P***(***MPC-MAA***) are shown in Fig. 1c, d. Relative intensities of nitrogen element are listed in Supplementary Table S1. Compared to ***IOL***, a strong and broad N1s peak at approximately 400 eV appeared on ***IOL-Plasma***, indicating that plasma treatment was achieved successfully^32^,^33^,^34^. After P(MPC-MAA) grafting, the peaks at 401.96 and 134 eV appeared on ***IOL-P***(***MPC-MAA**)*. These peaks corresponded to the -N-(CH_3_)_3_ and phosphate groups attributed to the MPC unit. Meanwhile, the peak at 400.04 eV corresponded to -NH-C(=O). These data indicate that P(MPC-MAA) was successfully grafted onto the IOL surface via the amidation reaction.

### Surface characterization of the IOLs

**Figure 2:**
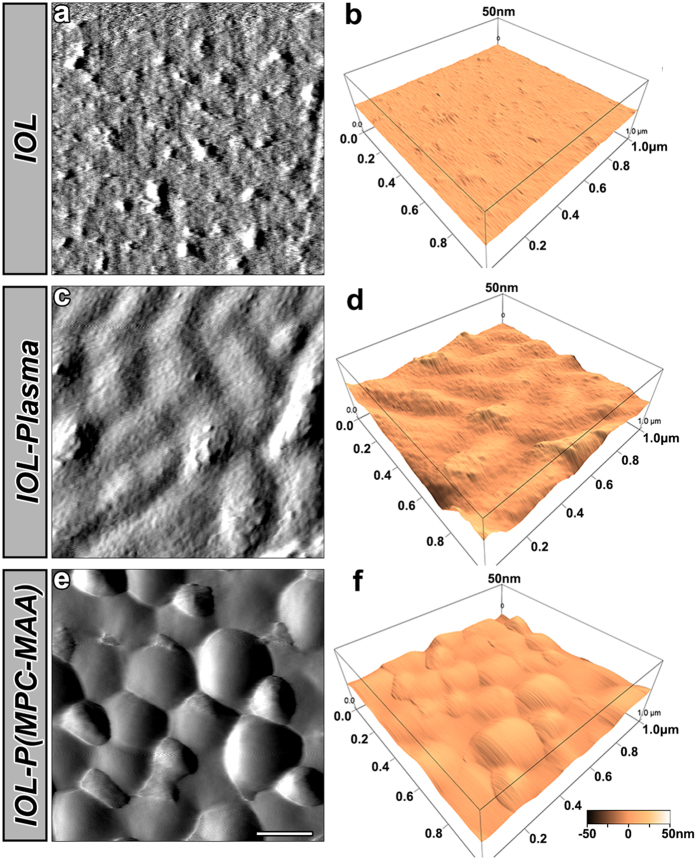
Surface characterization of IOL by AFM. Representative AFM images of the ***IOL**, **IOL-Plasma***, and ***IOL-P***(***MPC-MAA**)* surfaces. Area size for each scan: 1.0 × 1.0 μm^2^. Scale bar = 200 nm.

Surface topography affects protein adsorption and subsequent cell behaviors^35^. We first characterized the surface morphology of the samples by atomic force microscopy (AFM) (Fig. 2). ***IOL*** had a surface roughness of 0.787 nm, exhibiting a relatively even morphology with a few particles and shallow grooves (Fig. 2a,b). ***IOL-Plasma*** had many deep grooves appeared on the surface, and the roughness was increased to 4.818 nm (Fig. 2c,d). ***IOL-P** ( **MPC-MAA**)* exhibited many wave-like clusters of polymer chains, and the surface roughness was 3.469 nm (Fig. 2e,f).

**Figure 3:**
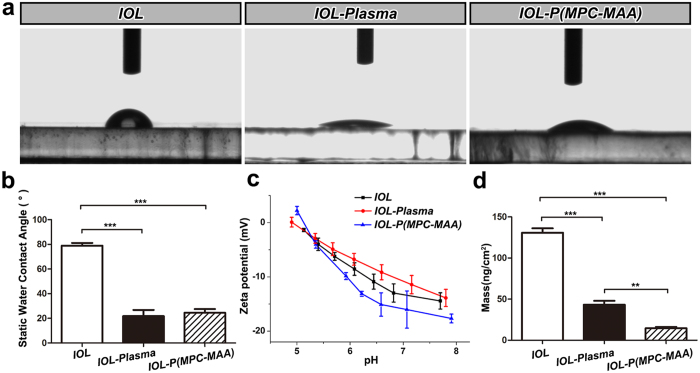
P(MPC-MAA) modification increases surface hydrophilicity and decreases protein adsorption. (**a**) Representative images of static WCA measurement in each group at 25 °C. (**b**) Quantification of static WCA in each group. n = 5. **P < 0.01, ***P < 0.001. (**c**) Quantification of zeta potential in each group. n = 4. (**d**) Measurement of BSA adsorption by QCM. n = 3. **P < 0.01, ***P < 0.001.

Next, we measured the water contact angles (WCAs) to characterize the hydrophilicity of the IOL surface (Fig. 3a,b). The WCA of ***IOL*** was 78.9 ± 2.2°, suggesting a hydrophobic surface property. Plasma treatment introduced amino groups onto the IOL surface, and the WCA of ***IOL-Plasma*** decreased to 21.8 ± 5.0°, indicating increased surface hydrophilicity. The WCA of ***IOL-P***(***MPC-MAA**)* also decreased to 24.5 ± 3.1°.

To investigate the electrokinetic properties of the samples, we measured the zeta potential of the samples (Fig. 3c). At pH 7.2, the zeta potential of ***IOL*** was −13.6 mv. The average zeta potential of ***IOL-Plasma*** increased to −11.5 mv due to the introduction of positively charged amino groups, while the average zeta potential of ***IOL-P***(***MPC-MAA**)* decreased to −16.4 mv due to the introduction of the negatively charged carboxylic acid groups.

The optical characteristics of the samples, such as diopter, resolution and transmission properties, demonstrated no significant differences between ***IOL-P***(***MPC-MAA**)* and ***IOL***. The haptics of all groups could endure bending and stretching 2.5 million times with a compression amplitude of +/−0.25 mm. These optical and physical properties meet the standards of State Food and Drug Administration (SFDA) in China.

### *IOL-P*(*MPC-MAA)* inhibits protein adsorption

Protein adsorption is the first phenomenon observed after IOL implantation, and will affect subsequent cell interaction in the material-tissue interface in the following minutes or hours^8^. We used bovine serum albumin (BSA) to monitor protein adsorption on the IOL surface by quartz crystal microbalance with dissipation (QCM-D) analysis (Fig. 3d). BSA adsorption on ***IOL*** was 130.8 ± 9.9 ng/cm^2^, which was similar to that on other hydrophobic IOL surfaces we previously reported^36^. Compared to ***IOL***, BSA adsorption on ***IOL-Plasma*** decreased to 43.1 ± 8.2 ng/cm^2^, and BSA adsorption on ***IOL-P***(***MPC-MAA**)* further decreased to 14.5 ± 3.1 ng/cm^2^. These data were consistent with the previous reports that increased surface hydrophilicity and introduction of negative charges onto the material surface can significantly decrease protein adsorption^16^,^17^.

### *IOL-P*(*MPC-MAA)* inhibits the adhesion and proliferation of lens epithelial cells *in vitro*

**Figure 4:**
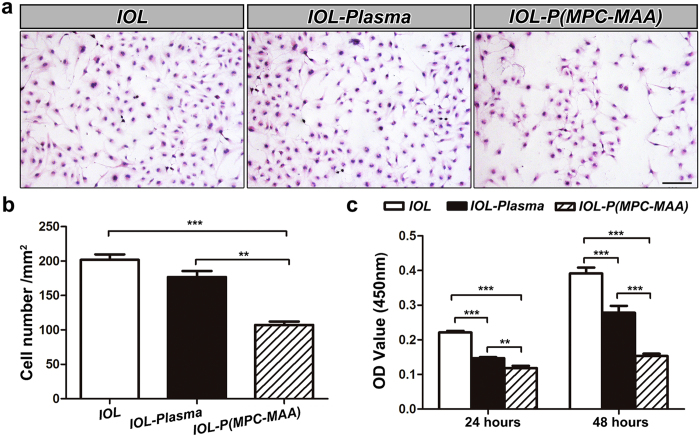
*IOL-P*(*MPC-MAA)* inhibits the adhesion and proliferation of LECs *in vitro*. (**a**) Representative inverted phase contrast microscope images of LECs attached to the surface of ***IOL**, **IOL-Plasma***, and ***IOL-P***(***MPC-MAA**)*, respectively. Scale bar = 100 μm. (**b**) Quantification of the number of cells attached to the surfaces of IOLs in (**a**). In each group, 5 IOLs were chosen, and in each IOL, five fields were selected with one in the central and four in peripheral quadrants at random. (**c**) Cell viability assay shows the proliferation of LECs on the surfaces of IOLs after incubation for 24 and 48 hours, respectively. n = 3. **P < 0.01, ***P < 0.001.

Cell interaction in the material-tissue interface include an initial phase of cell adhesion followed by subsequent cell proliferation and migration^12^. We used human lens epithelial cell (LEC) line SRA01/04 to evaluate cell behaviors on modified IOLs *in vitro*. Adhesion of LECs on ***IOL-P***(***MPC-MAA**)* (107.1 ± 5.1/mm^2^) was significantly decreased compared to that on ***IOL*** (201.7 ± 8.1/mm^2^) and ***IOL-Plasma*** (176.7 ± 8.9/mm^2^) (Fig. 4a,b). However, there was no significant difference between cell adhesion on ***IOL*** and ***IOL-Plasma** ( P* = 0.174). In order to characterize cell proliferation on the IOL surfaces, we incubated LECs on the IOL for 24 and 48 hours and performed a cell viability assay. Compared to ***IOL*** and ***IOL-Plasma**, **IOL-P***(***MPC-MAA**)* significantly decreased cell proliferation after 24 and 48 hours of incubation (Fig. 4c). Collectively, these results demonstrate that P(MPC-MAA) modification significantly increases cell repellency of the IOL surface.

### *IOL-P*(*MPC-MAA)* reduces postoperative inflammation

**Figure 5:**
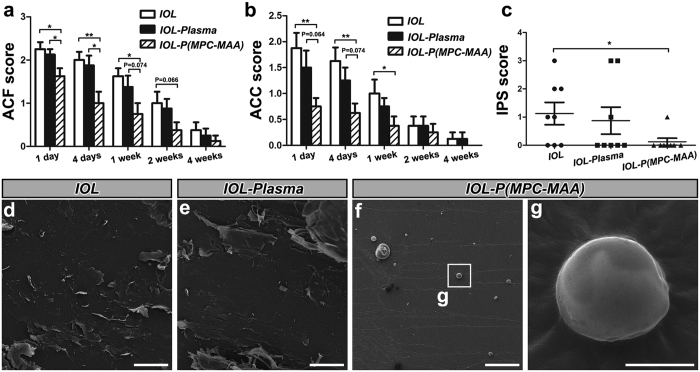
*IOL-P*(*MPC-MAA)* reduces postoperative inflammation after cataract surgery. (**a**,**b**) Quantification of ACF and ACC scores in each group 1 day, 4 days, 1 week, 2 weeks, and 4 weeks after surgery, respectively. n = 8. *P < 0.05, **P < 0.01. (**c**) Quantification of IPS scores in each group 8 weeks after surgery. n = 8. *P < 0.05, **P < 0.01. (**d**–**g**) Representative SEM images of ***IOL**, **IOL-Plasma***, and ***IOL-P***(***MPC-MAA**)* surfaces 8 weeks after surgery. (**d**–**f**) Scale bar = 100 μm. (**g**) Scale bar = 5 μm.

Uveal biocompatibility of the IOL can be assessed by the severity of postoperative inflammation^8^. Breakdown of the blood-aqueous barrier and the foreign body reaction to the IOL implant results in release of protein and cells into the anterior chamber, which can be manifested as anterior chamber flare (ACF) and anterior chamber cell (ACC) respectively^37^. Therefore, we first evaluated ACF and ACC scores as indicators of inflammation (Fig. 5a,b). Similar to the inflammatory responses in human patients after IOL implantation^4^,^38^, both ACF and ACC scores peaked 1 day postoperatively, and then decreased to the baseline after 4 weeks. Rabbit eyes with implantation of ***IOL-P***(***MPC-MAA**)* had significantly lower ACF and ACC scores 1 day, 4 days, and 1 week after surgery. Persistent inflammation may cause IPS, which refers to the adhesion of the iris to the anterior surface of the IOL or lens capsule. Eight weeks after surgery, slit lamp examination showed that ***IOL-P***(***MPC-MAA**)* implantation group had a significantly lower IPS score than ***IOL*** implantation group (Fig. 5c, Supplementary Fig. S3a). We also noticed other postoperative complications occurred in ***IOL**, **IOL-Plasma*** groups, including pupil capture (1 eye in ***IOL*** group and 1 eye in ***IOL-Plasma*** group), IOL displacement (1 eye in ***IOL*** group and 1 eye in ***IOL-Plasma*** group) and severe cortical proliferation (1 eye in ***IOL*** group) (Supplementary Fig. S3b). However, no obvious postoperative complication due to inflammation was found in ***IOL-P***(***MPC-MAA**)* implantation group. Intraocular pressure (IOP) values were within normal range in all the groups (Supplementary Fig. S4).

To further characterize the cellular response to the IOL implants, we extracted the IOLs 8 weeks after surgery and performed scanning electron microscopy (SEM). A large number of amorphous debris and polygonal cells were found adhered to the surfaces of ***IOL*** (Fig. 5d,e). However, only a few debris and small round cells were found on the surfaces of ***IOL-P***(***MPC-MAA**)* (Fig. 5f,g). Collectively, these results indicate that P(MPC-MAA) modification greatly improves uveal biocompatibility of hydrophobic acrylic IOLs *in vivo*.

### IOL-P(MPC-MAA) inhibits anterior capsule opacification

**Figure 6:**
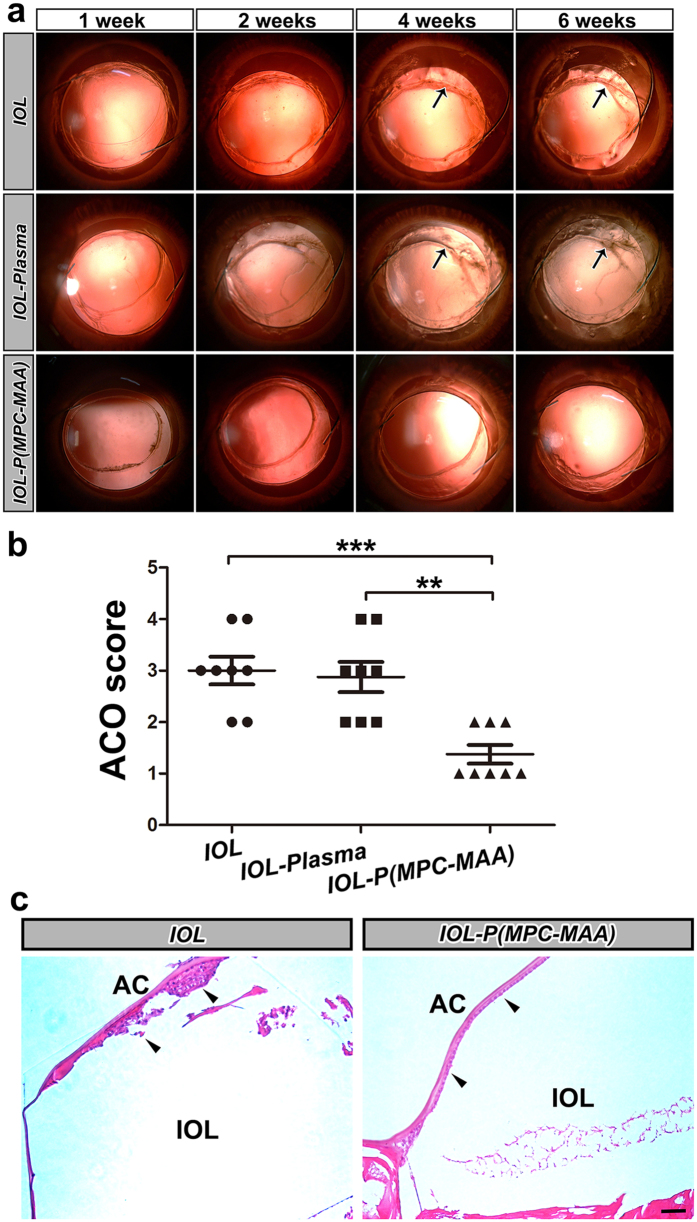
*IOL-P*(*MPC-MAA)* suppresses ACO formation after cataract surgery. (**a**) Representative retroillumination slit lamp photos of anterior capsule opacification in each group 1 week, 2 weeks, 4 weeks, and 6 weeks after surgery, respectively. Black arrows indicate anterior capsule fibrosis and shrinkage. (**b**) Quantification of ACO scores in each group 6 weeks after surgery. n = 8. **P < 0.01, ***P < 0.001. (**c**) Representative HE staining images of anterior capsule in each group 8 weeks after surgery. Black arrowheads indicate LECs under anterior capsule. AC: anterior capsule. Scale bar = 50 μm.

ACO is caused by proliferation and epithelial-mesenchymal transition (EMT) of the remnant LECs between the inner surface of the anterior capsule and IOL implant^39^. In our study, ***IOL*** and ***IOL-plasma*** implantation groups developed ACO 2 weeks after surgery (Fig. 6a). After 4 weeks, severe fibrosis occurred on the anterior capsule covering ***IOL*** and ***IOL-plasma*** optics, leading to anterior capsule shrinkage (black arrows). However, in ***IOL-P***(***MPC-MAA**)* implantation group, ACO developed slowly, and the anterior capsule was relatively transparent 6 weeks after surgery. The ACO score of ***IOL-P***(***MPC-MAA**)* group was significantly lower than that of the ***IOL*** and ***IOL-plasma*** groups 6 weeks postoperatively (Fig. 6b). Also, histopathological examination showed that multilayer LECs presented underneath the anterior capsule in ***IOL*** implantation group, while LECs were arranged regularly in a single layer in ***IOL-P***(***MPC-MAA**)* group (Fig. 6c, black arrowheads). The expression levels of EMT markers fibronectin (Fn) and α-smooth muscle actin (α-SMA) were lower in ***IOL-P***(***MPC-MAA**)* group compared to those in ***IOL*** group (Supplementary Fig. S5). TEM showed that in ***IOL*** group, LECs underneath the anterior capsule presented an elongated fibroblast-like appearance with massive ECM deposition (Supplementary Fig. S6a). However, in IOL-P(MPC-MAA) group, LECs maintained epithelial morphology with a few ECM depositions (Supplementary Fig. S6b). These results indicate that ***IOL-P***(***MPC-MAA**)* significantly suppressed LECs proliferation and EMT under the anterior capsule and thus inhibited ACO formation.

### IOL-P(MPC-MAA) does not affect posterior capsule opacification

**Figure 7:**
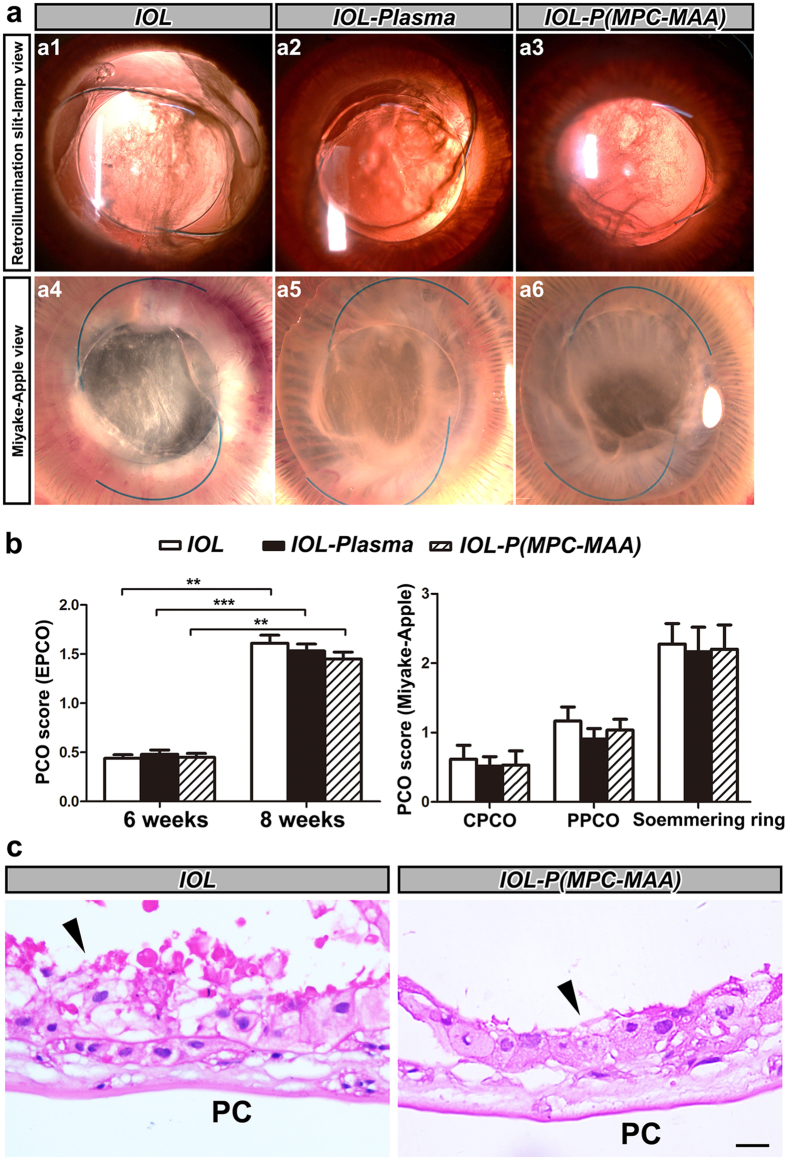
*IOL-P*(*MPC-MAA)* does not affect PCO formation after cataract surgery. (**a**) Representative photos of posterior capsule opacification in each group 8 weeks after surgery. a1–a3: retroillumination slit-lamp view, a4–a6: Miyake-Apple view. (**b**) Quantification of PCO scores in each group by EPCO 2000 software or Miyake-Apple view analysis, respectively. n = 8. **P < 0.01, ***P < 0.001. CPCO: central posterior capsule opacification, PPCO: periphery posterior capsule opacification. (**c**) Representative HE staining images of posterior capsule in each group 8 weeks after surgery. Black arrowheads indicate migration and proliferation of LECs on the posterior capsule. PC: posterior capsule. Scale bar = 50 μm.

PCO is caused by migration of remnant LECs to the posterior capsule^39^. In our study, slit lamp examination showed that all three groups developed moderate PCO 8 weeks after surgery (Fig. 7a). Fundus examination showed that the optic disk, retinal vessels and choroid vessels could not be clearly seen 8 weeks postoperatively (Supplementary Fig. S7). EPCO 2000 analysis showed no significant difference in all the groups, and Miyake-Apple view analysis showed no difference of CPCO, PPCO and Soemmering’s area in all the groups (Fig. 7b). In both ***IOL*** and ***IOL-P***(***MPC-MAA**)* groups, LECs exhibited fibroblast-like morphology and were arranged irregularly underneath the posterior capsule (Fig. 7c, black arrowheads) with massive ECM surrounded (Supplementary Fig. S6c,d). These results suggest that MPC-MAA surface grafting does not affect PCO formation.

## Discussion

Optimization of IOL biocompatibility is critical for vision reconstruction after cataract surgery. Uveal biocompatibility is typically important for hydrophobic acrylic IOL because many studies have shown that hydrophobic IOL will cause more inflammatory responses than hydrophilic IOL after implantation^3^,^40^,^41^. Both the cataract surgery and the IOL implant trigger the release and adhesion of inflammatory cells, including macrophages and giant cells, onto the IOL surface^8^, leading to a high incidence of IPS and ACO, especially in patients with blood-aqueous barrier damage. MPC has excellent biocompatibility since the phosphorylcholine group on MPC mimics the neutral phospholipids of the cell membrane^14^. Previous studies have shown that grafting MPC onto silicone IOL surface can reduce adhesion of macrophages^20^. However, grafting MPC monomers does not reduce aqueous flare *in vivo*, possibly due to inadequate negative charges on the material surface^14^. In this study, we synthesized the copolymer P(MPC-MAA) and covalently grafted this copolymer onto the surface of hydrophobic acrylic IOLs. Compared to MPC monomer, P(MPC-MAA) has two advantages. First, P(MPC-MAA) is heavily negatively charged (Fig. 3c). The introduction of negative charges by MAA resulted in a significant reduction of protein adsorption (Fig. 3d) and cell adhesion (Fig. 4). Second, the intermolecular repulsion between MAA in the copolymer could make more MPC diffuse into the aqueous humor, so that the IOL surface was more inert to the surrounding biological system. Therefore, P(MPC-MAA) modification significantly reduced post-operative inflammation after IOL implantation (Fig. 5a–c) and showed excellent biocompatibility *in vivo*.

The remaining anterior LECs (A cells) following cataract surgery have the potential to form fibrous tissue and cause capsular opacification around the capsulorhexis margin, resulting in ACO. Formation of ACO includes two stages: an early stage of LEC proliferation and a late stage involving EMT and ECM production. The LEC proliferation process is regulated by various cytokines and growth factors, such as IL-1, IL-6, transforming growth factor (TGF) and fibroblast growth factor (FGF), which are secreted by residual LECs and inflammatory cells^42^,^43^,^44^. Hydrophobic IOL has a higher incidence rate of ACO than hydrophilic IOL, because hydrophobic surfaces tend to attract more remnant LECs and inflammatory cells to adhere and proliferate^5^,^6^,^45^. Our results showed that surface modification by P(MPC-MAA) significantly suppressed ACO formation, which could be a direct consequence of decreased LECs adhesion and proliferation. Also suppression LECs and inflammatory cells adhesion may lead to less secretion of cytokines, contributing to a relatively mild inflammatory response in ***IOL-P***(***MPC-MAA**)* group than ***IOL*** and ***IOL-Plasma*** groups. This is consistent with other studies that hydrophilic surface modifications such as HSM coating^38^ or PEG-grafting^15^,^46^ could significantly reduce LECs adhesion and postoperative foreign-body reaction of hydrophobic IOL.

Posterior capsule opacification (PCO), also known as secondary cataract, results from proliferation, migration and EMT of residual LECs across the posterior capsule. In clinical application, hydrophobic IOL has a relatively low PCO rate compared to hydrophilic IOL, as the rapid adhesion of IOL to the posterior capsule can effectively inhibit the migration of LECs^12^,^47^. In this study, we did not observe a difference in PCO severity between ***IOL**, **IOL plasma*** and ***IOL-P***(***MPC-MAA**)* groups. Similarly, Xiaodan *et al*.^14^ also did not find a change in PCO incidence after grafting MPC on silicone IOL. It is possible that the surface property of IOL may be not as important as the optic configuration in the prevention of PCO. Many studies have shown that a sharp optic edge is the key factor for preventing LECs migration from anterior to the posterior capsule^48^,^49^,^50^. Although all the IOLs we used in this study had sharp optic edges, we still observed PCO formation in all the groups 8 weeks after surgery, possibly because rabbit LECs have higher proliferation and migration capacity than human LECs.

Interestingly, we noticed that introduction of amino groups by ammonia plasma treatment alone could also increase surface hydrophilicity, decrease protein adsorption and cell proliferation. However, our previous study showed that the increased hydrophilicity of IOL after plasma treatment can only last for 14 days^51^. On the contrary, covalent immobilization of hydrophilic molecules onto the material surface can greatly weaken the hydrophobic recovery process^20^,^46^. Here, we showed that although the hydrophilicities of ***IOL-Plasma*** and ***IOL-P***(***MPC-MAA**)* were comparable after modification, ***IOL-P***(***MPC-MAA**)* exhibited more protein resistance. Moreover, only ***IOL-P***(***MPC-MAA**)* showed decreased postoperative inflammation and ACO formation *in vivo*. Therefore, modification by plasma treatment alone is insufficient for the improvement of IOL biocompatibility.

In conclusion, we synthesized a new copolymer P(MPC-MAA) and successfully grafted the copolymer onto the surface of hydrophobic acrylic IOL by plasma technology. ***IOL-P***(***MPC-MAA**)* showed increased surface hydrophilicity and reduced protein adsorption while maintaining the bulk optical and physical properties. ***IOL-P***(***MPC-MAA**)* significantly inhibited LECs adhesion and proliferation *in vitro*. and suppressed postoperative inflammation and ACO formation in *vivo*. Overall, these results suggest that P(MPC-MAA) modification improved uveal and capsular biocompatibility of hydrophilic acrylic IOLs. More studies need to be carried out to assess the long-term biocompatibility of ***IOL-P***(***MPC-MAA**)*.

## Methods

### Synthesis and purification of P(MPC-MAA)

0.01 mol MPC (Nanjing Institute of Natural Science and Technology Development, Nanjing, China) and 0.01 mol MAA (Kemiou Chemical Reagent Co., Ltd, Tianjing, China) were dissolved in 20 g of ultrapure water (monomer concentration: 1 mol/L). After argon was introduced for 30 minutes, a sodium sulfite/ammonium sulfate initiator system (ammonium persulfate, sodium sulfite = 1:1.5) was added at a concentration of 0.03 mol/L. The reaction was carried out at 37 °C for 24 hours and was stopped with liquid nitrogen. After introducing anhydrous ethanol, sedimentation was carried out and collected by a suction filter. The sediment was again dissolved in water and dialyzed for 3 days. Then, the sample was freeze-dried for 2 days, and P(MPC-MAA) was obtained. FT-IR spectra was obtained using an FT-IR analyzer (VECTOR-22, Bruker, Germany) with the potassium bromide pressed-disk technique for 32 scans over the 500–4,000 cm^−1^ range at a resolution of 4.0 cm^−1^. The composition of the polymers was determined by ^1^H NMR (AVANCE 300, Bruker, Germany) spectral measurements at 400 MHz.

### P(MPC-MAA) grafting

Hydrophobic acrylic IOLs (EYEGOOD Medicals Co., Ltd, Zhuhai, China) were treated with ammonia plasma in a DL-01 model plasma generator (Omega Machinery Electronic Technology Co. Ltd., Suzhou, China) with power at 80 W for 15 minutes. P(MPC-MAA) solution was obtained containing EDC and HOBt (1-hydroxy-benzotriazole) at the ratio of 1:1.5:2 (-COOH:EDC:HOBt). IOLs were immersed in the 2.5% P(MPC-MAA) solution overnight, and dried under nitrogen flow. IOLs for cell assays and *in vivo* implantation were autoclaved with ethylene oxide, sealed in a sterilized package and stored at room temperature.

### X-ray photoelectron spectroscopy(XPS)

XPS was measured by a photoelectron spectrometer (AXIS ULTR DLD, Kratos, England) using an Al Kα (1486.4 eV) monochromatic X-ray source at a pressure of 2 × 10^−9^ Torr and a scan area of 0.7 × 0.3 mm^2^. The high resolution scanning of elemental spectra was performed at 40 eV pass energy.

### Atomic force microscopy(AFM)

Surface topography was tested with an atomic force microscope (MFP-3D-S, Asylum Research, USA). AFM imaging was performed under dry conditions at room temperature (24 ± 2 °C). Samples were analyzed over a 1.0 × 1.0 μm region at a resolution of 512 × 512 pixels. Root mean square roughness values were determined from height retrace images for each sample.

### Static water contact angle (WCA) measurement

WCA was characterized with a contact angle goniometer (OCA15, Dataphysics, Germany) at 25 °C using distilled water as a reference liquid. A total of 1.00 μL of reference liquid was pumped onto the surface through a stainless steel needle at a rate of 1.0 μL/s. The results are mean values calculated from five independent measurements on different points of the films.

### Zeta potential measurement

Zeta potential was obtained using an electrokinetic analyzer (SurPASS, Anton Paar Surpass, Austria). For the determination of zeta potential, streaming current measurements were performed using an Adjustable Gap Cell (SurPASS, Anton Paar Surpass, Austria). 1.0 mM potassium chloride (KCl) solution was used as the background electrolyte, and 0.1 M potassium hydroxide as well as 0.1 M hydrochloric acid solutions were used to adjust the pH value to 7.2.

### Optical and physical characteristics

Diopter, resolution, transmission properties and anti-fatigue resistance of the IOL haptics were assessed according to the standards of State Food and Drug Administration (SFDA) by Medical Equipment Quality Supervision and Inspection Center of Zhejiang Province in China.

### BSA adsorption assay

BSA adsorption of the surface was measured by quartz crystal microbalance with dissipation (QCM-D, E4, Q-Sense, Sweden). Briefly, the BSA solution (dissolved in PBS buffer at a concentration of 50 μg/mL) was introduced onto the samples. After balancing, PBS was introduced again to wash off the non-adsorbed protein. Then, BSA adsorption was obtained from Q-Tools.

### Cell adhesion assay

IOLs were placed into a 48-well plate. 300 μL SRA01/04 cell (human lens epithelial cell) suspension at a concentration of 1 × 10^4^/mL was loaded onto the IOL surface. After incubation for 12 hours, the IOLs were stained with hematoxylin and eosin (HE) and examined with an inverted phase contrast microscope (CKX41, Olympus, Japan). Five fields were selected with one in the central and four in peripheral quadrants at random. Image-Pro Plus 6.0 was used to quantify the number of LECs in each field. At least 5 IOLs in each group were tested.

### Cell viability assay

The seeding procedure was the same as described above. After incubation for 24 or 48 hours, 200 μL Dulbecco’s Modified Eagle Medium (DMEM) with 10% fetal bovine serum (FBS) and 20 μL Cell Counting Kit-8 (CCK-8) reagent were added to IOLs. Wells without IOLs were used as controls. After incubation for 1 hour, the OD values at 450 nm were measured with a microplate reader. The assay was repeated 3 times.

### Phacoemulsification and IOL implantation

Twenty-four 1.5 kg male New Zealand albino rabbits were divided into three groups at random. Phacoemulsification was performed on the left eye using the Legacy 20000 System (Alcon Laboratories, Fort Worth, TX, USA). Briefly, a 3.2-mm corneal limbus tunnel incision was made at the 12 o’clock position, followed by a central continuous curvilinear capsulorhexis with 5.5 mm in diameter. Then, the lens materials were extracted and IOL was implanted into the capsule. The tunnel incision was closed with interrupted 10-0 nylon sutures. All surgeries were performed by one surgeon (M.X.W.), who was blind to the group assignment. All experiments were conducted in accordance with the ethical guidelines set forth by the Laboratory Animal Care and Use Committee of the Association for Research in Vision and Ophthalmology (ARVO). The study protocol was reviewed and approved by the Animal Ethics Committee of Zhongshan Ophthalmic Center, Sun Yat-sen University, China.

### Follow-up ophthalmic examinations

Digital slit lamp photos were taken by the SL-D7 anterior eye segment analysis system (Topcon Medical Systems, Inc., Tokyo, Japan) at indicated times postoperatively. Intraocular pressure (IOP) was measured by a Tono-Pen tonometer (Reichert Inc., Seefeld, Germany). Fundus images were acquired by a fundus camera (Topcon Medical Systems, Inc., Tokyo, Japan). All examinations were conducted by two researchers who were blind to the group assignment. Serious PCO usually occurred at 8 weeks due to the strong proliferative ability of rabbit LECs, so we defined 8 weeks postoperatively as the endpoint of the ophthalmic examinations.

### Inflammation evaluation

Anterior chamber flare (ACF), anterior chamber cell (ACC), iris posterior synechiae (IPS) were scored to evaluate uveal biocompatibility of the IOL as previously described^52^. The grading is summarized in Supplementary Table S2. Postoperative complications, such as corneal edema, glaucoma, IOL displacement, pupil capture, and cortical proliferation, were also recorded.

### ACO scoring

Six weeks after surgery, ACO was scored from grade 0 to IV based on the severity of the anterior capsule opacity and the contraction of the anterior capsulorhexis opening: Grade 0: clear (transparent) anterior capsule; Grade I: opacification localized at the edge of the capsulorhexis; Grade II: moderate and diffuse opacification, in some cases with areas of capsular folding; Grade III: intense opacification, with areas of capsular folding; Grade IV: constriction (phimosis) of the capsulorhexis opening^45^.

### PCO scoring

PCO was quantified by Evaluation of Posterior Capsule Opacification (EPCO) 2000 software or Miyake-Apple view analysis. Standard retroillumination pictures were taken 6 and 8 weeks postoperatively, imported into EPCO 2000 and processed as previously described^53^.

Eight weeks after surgery, the rabbits were euthanatized and the eye balls were enucleated for Miyake-Apple view analysis. Eye balls were sectioned at the equator and gross examinations were performed from the posterior aspect. Miyake-Apple view analysis of PCO was conducted as previously described^54^.

### Hematoxylin-eosin (HE) staining

Three capsules with IOLs in each group were fixed in 10% formalin for 2 hours, dehydrated and embedded in paraffin. Sections were cut on a microtome (RM2235, Leica, Germany) at 4 μm and stained with HE.

### Immunofluorescent staining

Three capsules without IOLs in each group were embedded and stored at −80 °C. Sections (8 μm) were cut using a cryostat, fixed in cold acetone for 10 min, incubated with 0.2% Triton X-100 for 10 min, and blocked with 1% BSA. Sections were then incubated with primary antibodies at 4 °C overnight, and incubated with secondary antibody for 1 hour at room temperature. The sources and dilutions of antibodies are: mouse anti-fibronectin (1:100, ab6328, Abcam, MA, USA), mouse anti-α-SMA (1:100, ab7817, Abcam), Alexa Fluor 488-conjugated anti-mouse IgG (1:1000, #4408, Cell Signaling Technology, MA, USA) and Alexa Fluor 555-conjugated anti-mouse IgG (1:1000, #4409, Cell Signaling Technology).

### Scanning electron microscopy(SEM)

Five IOLs in each group were extracted, fixed in 2.5% glutaraldehyde, and post-fixed in 1% osmium tetroxide. Dehydration was carried out through a graded ethanol series at room temperature. After critical drying point, the IOLs were sputter-coated with gold and examined using a scanning electron microscope (Quanta 200, FEI, Hillsboro, Oregon, USA).

### Transmission electron microscopy (TEM)

Two capsules without IOLs were fixed in 2.5% glutaraldehyde, post-fixed in 1% osmium tetroxide (w/v 0.1 M phosphate buffer), dehydrated through a graded ethanol series and embedded in resin. About 80 nm ultrathin sections were obtained and stained with uranyl acetate and lead citrate, and examined by a transmission electronic microscope (Tecnai G2 Spirit Twin, FEI, Hillsboro, Oregon, USA).

### Statistical analysis

SPSS 17.0 software package (SPSS Inc., Chicago, IL, USA) was used for statistical analysis. All data are represented as means ± S.D. Kruskal-Wallis test was used to analyze the ACC, ACF, IPS, ACO and PCO scores. One-way analysis of variance (ANOVA) followed by Bonferroni’s post hoc test was used to compare means of the other results. All statistical tests were two tailed. *P* < 0.05 was considered statistically significant.

## Additional Information

**How to cite this article**: Tan, X. *et al*. Improvement of Uveal and Capsular Biocompatibility of Hydrophobic Acrylic Intraocular Lens by Surface Grafting with 2-Methacryloyloxyethyl Phosphorylcholine-Methacrylic Acid Copolymer. *Sci. Rep.* **7**, 40462; doi: 10.1038/srep40462 (2017).

**Publisher's note:** Springer Nature remains neutral with regard to jurisdictional claims in published maps and institutional affiliations.

## Supplementary Material

Supplementary Information
