# Supplementary material for: Improvement of Uveal and Capsular Biocompatibility of Hydrophobic Acrylic Intraocular Lens by Surface Grafting with 2-Methacryloyloxyethyl Phosphorylcholine-Methacrylic Acid Copolymer
Source: Sci Rep. 2017 Jan 13;7:40462. doi: 10.1038/srep40462 (PMC5234006; doi:10.1038/srep40462)
Supplement: Supplementary Information [file srep40462-s1.pdf]

Supplementary information

Improvement of Uveal and Capsular Biocompatibility of Hydrophobic Acrylic Intraocular Lens by Surface Grafting with 2-Methacryloyloxyethyl Phosphorylcholine-Methacrylic Acid Copolymer

Xuhua Tan [1#], Jiezhao Zhan [2,3 #], Yi Zhu [1], Ji Cao [3,5], Lin Wang [2,3], Sa Liu [2,3], Yingjun Wang [2,3], Zhenzhen Liu [1], Yingyan Qin, Mingxing Wu [4*], Yizhi Liu [1*] & Li Ren [2,3*]

[1] State Key Laboratory of Ophthalmology, Zhongshan Ophthalmic Center, Sun Yat-sen University, Guangzhou, Guangdong, 510060, China

[2] National Engineering Research Center for Human Tissue Restoration and Reconstruction, South China University of Technology, Guangzhou, Guangdong, 510006, China

[3] School of Materials Science and Engineering, South China University of Technology, Guangzhou, Guangdong, 510641, China

[4] Department of Cataract, Zhongshan Ophthalmic Center, Sun Yat-sen University, Guangzhou, Guangdong, 510060, China

[5] EYEGOOD Medicals Co., Ltd, Zhuhai, Guangdong, 519085, China

[#] These authors contributed equally to this work.

[*] Correspondence and requests for materials should be addressed to L.R. (email: psliren@scut.edu.cn), Y.L. (email: yizhi_liu@aliyun.com) or M.W. (email: wumingx@mail.sysu.edu.cn)

Figure S1. The chemical structures of **(a)** 2-methacryloyloxyethyl phosphorylcholine (MPC), **(b)** methacrylic acid monomer (MAA), and **(c)** P(MPC-MAA).

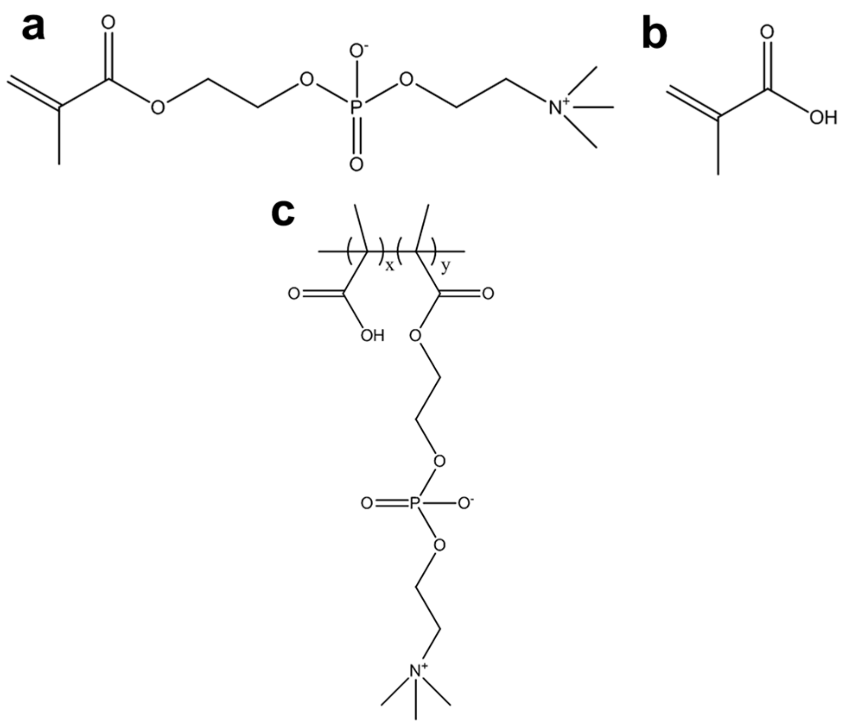

Figure S2. Molecular weight (MW) and polydispersity index (PDI) values of P(MPC-MAA) determined by GPC in aqueous sodium nitrate (0.1 M with 0.02 wt % of NaN_3) solution calibrated with poly (ethylene oxide) standards.

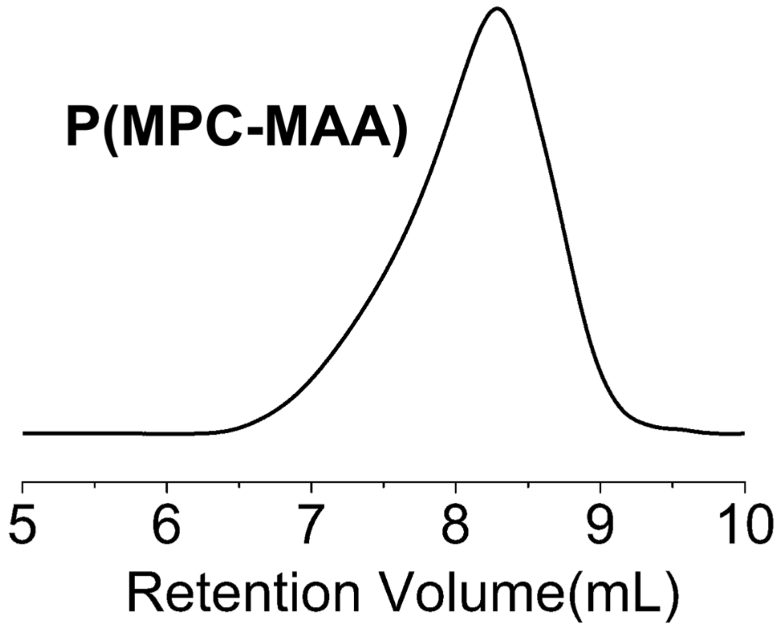

Figure S3. *IOL-P(MPC-MAA)* reduces postoperative inflammations after cataract surgery. (a) Representative slit lamp photos indicate the development of IPS in all groups. ***IOL*** and ***IOL-plasma*** developed Grade III (a1) and Grade I (a2) IPS, while no IPS was found in most ***IOL-P(MPC-MAA)*** (a3). Black arrowheads indicate adhesion of iris onto the anterior capsule or IOL surface. **(b)** Representative slit lamp photos indicate the postoperative complications developed in ***IOL*** and ***IOL-plasma*** groups, including pupil capture (b1, white arrowhead), severe cortical proliferation (b2, white arrowhead) and IOL displacement (b3, white arrowhead).

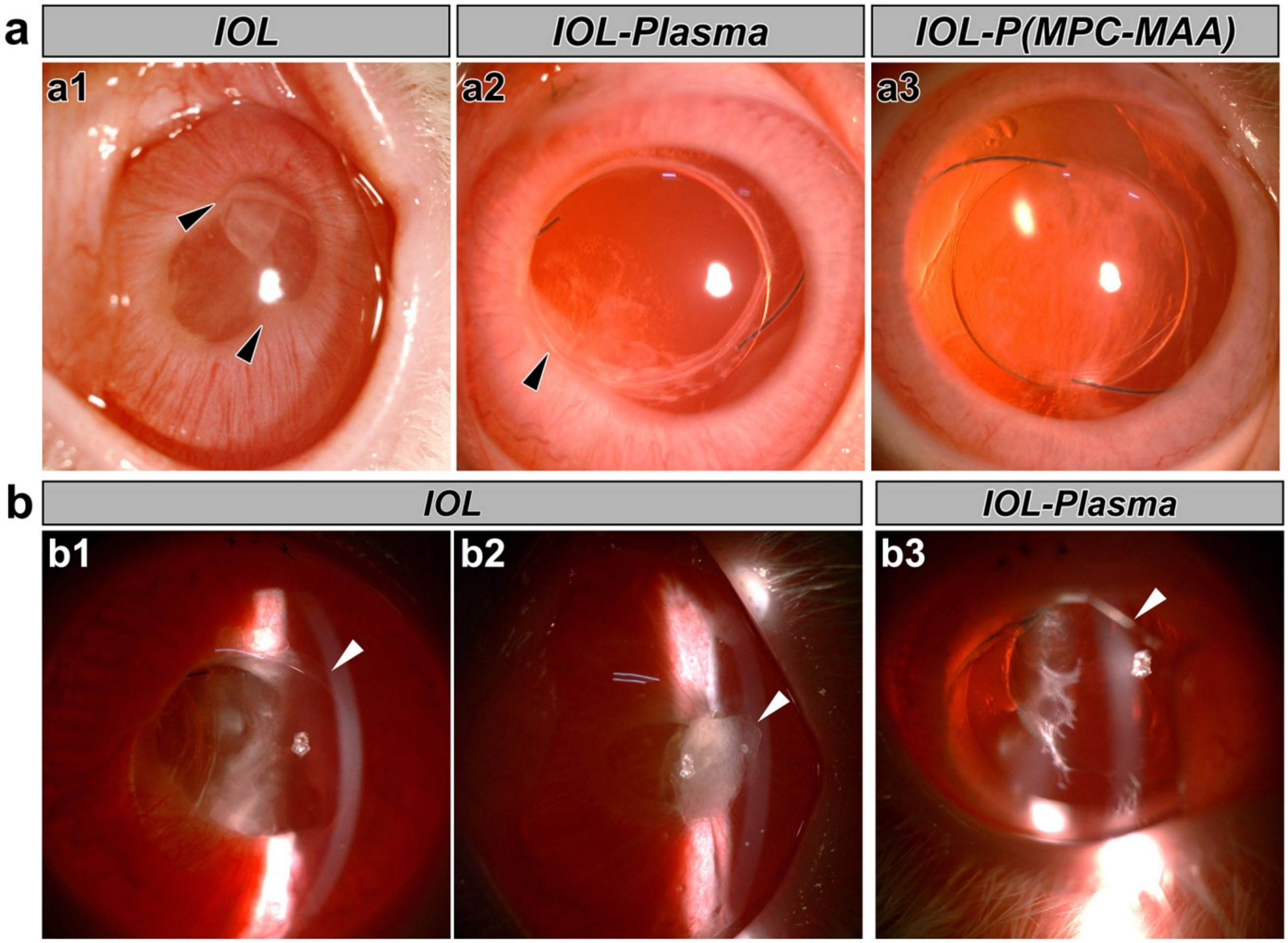

Figure S4. IOP was comparable in all the groups after cataract surgery. IOP was measured preoperatively and 1 day, 4 days, 1 week, 2 weeks and 4 weeks postoperatively. n=3.

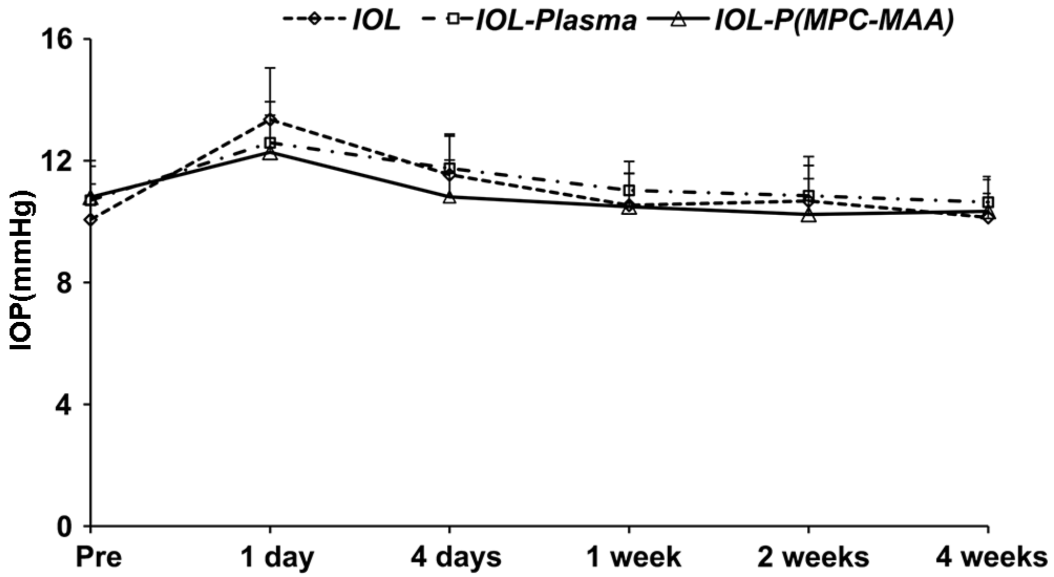

Figure S5. *IOL-P(MPC-MAA)* reduces EMT of LECs under anterior capsule. Anterior capsules were stained with Fn (green), α-SMA (red) and DAPI (blue) in ***IOL*** and ***IOL-P(MPC-MAA)*** groups 8 weeks after surgery. Scale bar: 20μm.

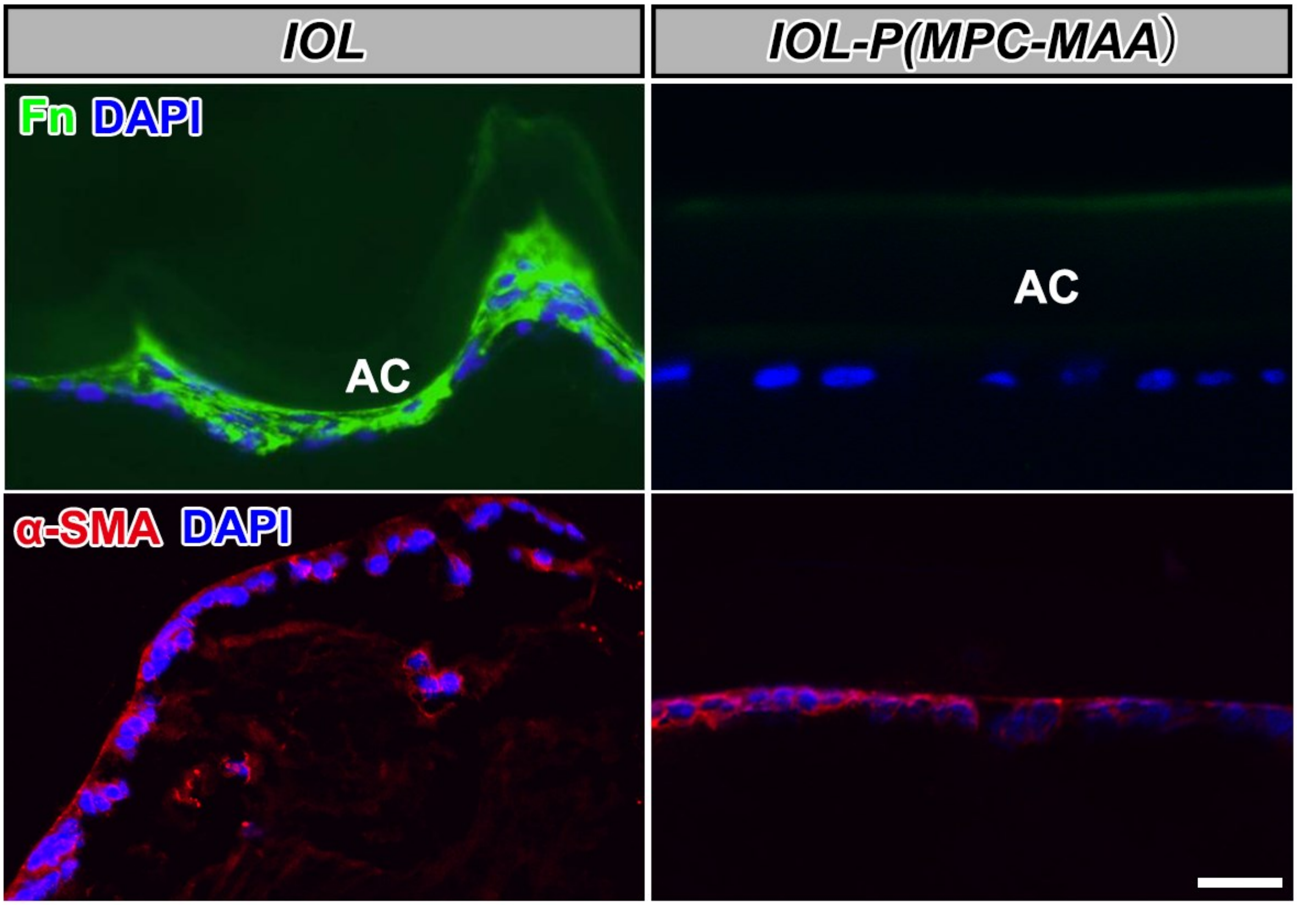

Figure S6. Representative TEM images of anterior capsules **(a-b)** or posterior capsules **(c-d)** in ***IOL*** and ***IOL-P(MPC-MAA)*** implantation groups 8 weeks after surgery. White arrowheads indicate deposition of ECM. AC: anterior capsule, PC: posterior capsule, n: nucleus.

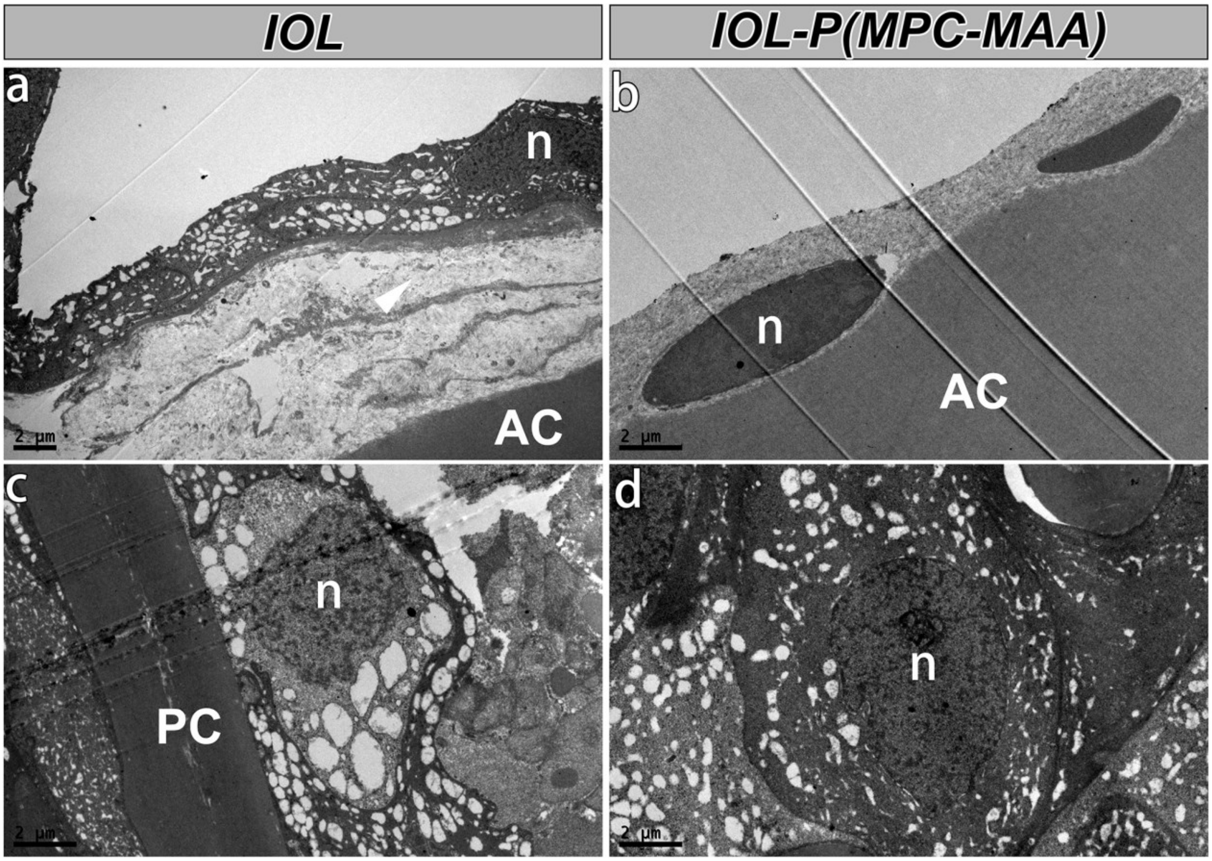

Figure S7. Representative fundus images in each group 8 weeks after surgery. Black arrowheads indicate rabbit optic disk.

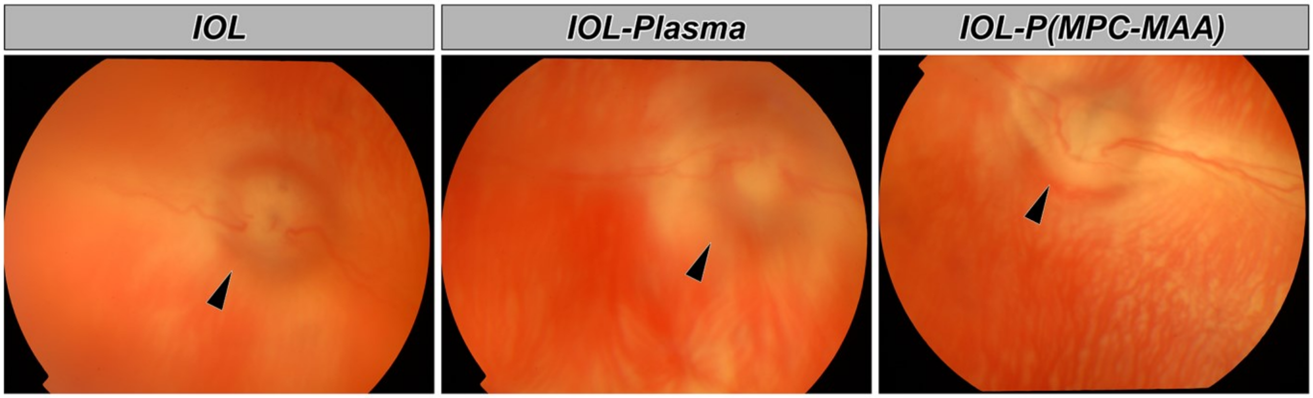

Table S1. Relative intensities of the N1s peaks of the IOLs.

	Position(eV)	Relative intensity (%)	Possible element state
IOL	399.38	100	N-C
IOL-Plasma	399.46	100	N-C
	399.19	26.31	N-C
IOL-P(MPC-MAA)	400.04	17.34	N-C(=O)
	401.96	56.34	N-C(CH_3)

Table S2. Anterior chamber inflammation assessment after surgery.

	Anterior Chamber Flare	Anterior Chamber Cells	Iris Posterior Synechiae
Grade	Flare Count	Cell Numbers	Synechia Range
0	no	0	no
I	Slight (barely detectable)	1-15	<1 quadrant
II	Moderate (iris and lens clear)	16-25	≤2 quadrants
III	Marked (iris and lens hazy)	26-50	≤3 quadrants
IV	Intense (fibrin clot)	> 50	>3 quadrants

Notes: Slit lamp biomicroscopy parameters: 16× magnification; 1×1 mm oblique high-intensity beam; aimed at central cornea in the pupillary axis; focus in the anterior aqueous humor; first count of cells performed at the plane of focus. The scores were evaluated by the same examiner and repeated 3 times.
